# The use of e-cigarettes among university students in Malaysia

**DOI:** 10.18332/tid/99539

**Published:** 2018-12-10

**Authors:** Sharifa Ezat Wan Puteh, Roslina Abdul Manap, Tidi Maharani Hassan, Izzah Syazwani Ahmad, Idayu Badilla Idris, Fariza Md Sham, Andrea Ban Yu Lin, Chun Ian Soo, Rashidi Mohamed Pakri Mohamed, Ahmad Irdha Mokhtar, Hazli Zakaria, Jing Lee, Amer Siddiq Amer Nordin, Suthahar Ariaratnam, Mohd Zaliman Mohd Yusoff

**Affiliations:** 1 Department of Community Health, Faculty of Medicine, Universiti Kebangsaan Malaysia Medical Centre, Kuala Lumpur, Malaysia; 2 Respiratory Units, Faculty of Medicine, Universiti Kebangsaan Malaysia Medical Centre, Kuala Lumpur, Malaysia; 3 Department Da’wah and Leadership Studies, Faculty of Islamic Studies, Universiti Kebangsaan Malaysia, Selangor, Malaysia; 4 Department of Family Medicine, Faculty of Medicine, Universiti Kebangsaan Malaysia Medical Centre, Kuala Lumpur, Malaysia; 5 University Malaya Centre of Addiction Sciences (UMCAS), University Malaya, Kuala Lumpur, Malaysia; 6 Department of Psychiatry, Faculty of Medicine, Universiti Kebangsaan Malaysia Medical Centre, Kuala Lumpur, Malaysia; 7 Institute for Environment and Development (LESTARI), Universiti Kebangsaan Malaysia, Selangor, Malaysia; 8 Department of Psychological and Behavioural Medicine, Faculty of Medicine, Universiti Teknologi MARA (UiTM), Selayang Campus, Batu Caves, Selangor, Malaysia; 9 Department of Software Engineering, College of Information Technology, Universiti Tenaga Nasional (UNITEN), Kajang, Malaysia

**Keywords:** electronic cigarettes, smoking, university, students, Malaysia

## Abstract

**INTRODUCTION:**

E-cigarette use is an emerging phenomenon with increasing recognition and acceptance globally. This study aims to create a profile of e-cigarette users among university students in Malaysia.

**METHODS:**

The study was conducted using a cross-sectional research involving six universities in Malaysia. A semi-structured questionnaire was distributed to 1302 randomly selected students, who either smoked cigarettes and/or e-cigarettes. The 2011 version of Global Adult Tobacco Surveys (GATS) tool was used to record the respondents’ sociodemographic data.

**RESULTS:**

The study revealed that 74.9% of the respondents smoked e-cigarettes; 40.3% used both cigarettes and e-cigarettes (dual users), and 34.5% were exclusive e-cigarette users. The exclusive use of e-cigarettes was related to gender (OR=0.18, 95% CI: 0.09–0.39). Also, male respondents were the majority users (95%). Of the respondents, 75.2 % were Malays, 98.0% single and most believed they have no health problems (92.1%). Further findings revealed the occurrence of adverse effects, dizziness 14.4%, cough 14.1%, and headaches 12.4%. Overall, 57.8% of the respondents used e-cigarettes as a smoking cessation tool, while others consider e-cigarettes a self-image enhancing tool or as part of social activities.

**CONCLUSIONS:**

Further research on the use of e-cigarettes should be conducted on a large number of respondents in other settings to augment the findings of this study, and also guide policy making on and prevention practice of e-cigarette use, among the general student population in Malaysia.

## INTRODUCTION

The electronic-cigarette (e-cigarette) is an electronic device, also known as vape, containing a cartridge filled with liquid nicotine and/or other chemicals, producing inhalable smoke. E-cigarette use has quickly gained popularity worldwide^1^, especially among current and former smokers^2-6^. E-cigarette manufacturers promote it as a safer, cheaper and an alternative product for smoking cessation. As the popularity and use increase, so is concern about public health. In 2009, Australia, Brazil, China, Uruguay^7^, Brunei, Cambodia, Indonesia, Singapore, Thailand, and Vietnam^8^ completely banned the sale and marketing of e-cigarettes; while New Zealand, United Kingdom, and other European countries allowed marketing of e-cigarettes. South– East Asia is one of the regions in the world with the highest number of countries (6 out of 11 countries in the region) that banned the use of e-cigarettes^8^. However, some aspects of the banned regulations in these countries need improvement^9^.

The International Tobacco Control Policy Evaluation Project (ITC Project) reports the use of e-cigarettes in 10 countries, through a survey conducted between 2009 and 2013. Malaysia was found to be the country with the highest prevalence of users, at 14%, while other countries like Republic of Korea and Australia had 7% each, United States 6%, United Kingdom 4%, Netherlands 3%, Canada 1%, and China 0.05%^1^. The 2015 Global Adult Tobacco Survey (GATS) conducted in the South-East Asian (SEAN) region, gave the following e-cigarette use prevalence for countries in the region: Indonesia 0.3% and Malaysia 0.8%, in 2011^10^; Philippines 1.7%^11^ and Vietnam 0.2%^12^. Also, the Global Youth Tobacco Survey (GYTS) of 2016 showed the prevalence of e-cigarette use among students (age 13–15 years) to be 2.3% in Cambodia^13^ and 5.7% in Myanmar^14^. Noteworthy, the survey revealed a 3.2% prevalence rate for Malaysia^1,14,15^, which makes it the largest vaping market in the SEAN region^16^.

Wong et al. reported 39.9% of e-cigarette use among young students of higher institutions and 36% use among young professionals in their study conducted in the Selangor and Kuala Lumpur areas of Malaysia^17^. In an attempt to check the menace of e-cigarette use, the Higher-Education Minister of Malaysia announced in November 2015 a ban on e-cigarette use and tobacco smoking in universities. The ban is in effect in several Malaysian universities^18,19^.

E-cigarette use prevalence is high among the young, and diverse reasons for the use have been reported in several studies. Some studies state that cigarette (tobacco) smoking is an important associated factor of e-cigarette use among young people^20-23^, while the older users consider it an alternative to tobacco smoking^24,25^. Other reasons for the use are: experimenting due to curiosity^26-29^, interesting flavours^30,31^, ‘just for fun’^29,32^, popularity^33^ and ‘just experimenting’^34^. The risk associated with e-cigarette use is said to be much less than that of tobacco smoking. If the claim is right, the harms related to tobacco smoking would be substantially reduced, with benefits for cardiovascular health^35^. However, more research needs to be conducted to ascertain the effects of e-cigarette use compared with tobacco smoking.

Several aspects of e-cigarette use in Malaysia were reported in various studies, but none was on the users’ profile. As such, this study aims to explore the factors associated with e-cigarette use, in order to create e-cigarette users’ profile based on sociodemographic distribution, source of information and supply, gender, and adverse effects, amongst other features, in six Malaysian universities.

## METHODS

### Participants and procedure

The study was conducted through a cross-sectional field survey research approach, from August 2016 to December 2016. Six out of sixteen universities situated in Klang Valley, Malaysia, were randomly selected; three public and three private universities. As prevalence for e-cigarette users among adults in Malaysia was 14%^1^, and the total population was estimated at 204000 students, a sample size of n=185 (95% Confidence Interval (CI) and 5% significant level) from each of the selected universities was obtained using the Leslie Kish formula. A total of 1302 participants were chosen through a box-model random sampling technique.

Inclusion criteria of this study were: 1) university student, 2) age 18 years and older, and 3) student currently smoking a conventional cigarette or use of e-cigarette at the time of the study. Exclusion criteria were: 1) student on leave and/or not attending classes for three months, 2) student suspended by university, 3) student did not wish to participate in the study, and 4) student with cognitive disorders due to diseases like dementia, Parkinson’s disease or schizophrenia.

### Measurements

The questionnaire, in English and Malay, contained both closed and open-ended questions, divided into three sections: 1) sociodemographic background and current health status; 2) smoking history; and 3) e-cigarette use. The sociodemographic section included age, gender, race/ethnicity, educational levels, nationality, marital status, total household income and health problems. Questionnaires were distributed to the participants by the lead researcher and a trained research assistant. Ethical approval for the study was granted by the Secretariat of Research and Innovation, Universiti Kebangsaan Malaysia Medical Centre (Code: FF-2016-301) and the administrative board of each participating university. Information sheets and consent forms were distributed to all students, and active consent was received from the participants. The respondents were duly informed that participation in the study was voluntary, and that their identity will remain anonymous. They were also informed that a response to a question was neither ‘right’ nor ‘wrong’.

Questions on smoking history were adapted from the GATS 2011 with slight modification to suit the study. Participants were asked if they had ever smoked a cigarette, smoking habits of their immediate family members, age at smoking initiation, amount of smoking and type of cigarette used, among other questions. Questions on the reasons for e-cigarette use were based on 28 items and a 5-point Likert scale (1=totally disagree, 2=disagree, 3=unsure, 4=agree, 5=totally agree). Responses were coded in two categories: low (total score lower than mean score) and high (total score higher than mean score).

### Statistical analysis

The collected data were analyzed using SPSS version 23.0, using frequencies (n), percentages (%), chi-square tests and multivariable binary logistic regression models. Level of significance was set at p<0.05 and regression results are presented as Adjusted Odds Ratios (AOR) with 95% Confidence Interval (CI).

## RESULTS

### Participants’ background

The respondents were between the ages of 18 to 40 years: males n=1234 (94.8%) and females n=68 (5.2%); mean age and standard deviation of 21.15 ± 2.55 years; ethnic Malay 75.2%, Malaysian nationals 97.9%, and single 98.0%. Distribution based on education level was PhD/DrPH 0.6% and undergraduate 65.2%. The household mean monthly income was MYR 5760.89 ± 7411.14, and the propotion of participants with a self-perception of being healthy was 92.1% (Table 1).

**Table 1:** Distribution of sociodemographic characteristics of respondents (n=1302 )

*Variables*	*Frequency (n)*	*Per cent (%)*
**Age (years)**		
18–20	376	28.9
21–25	880	67.6
26–30	38	2.9
>30	8	0.6
**Gender**		
Male	1234	94.8
Female	68	5.2
**Ethnicity**		
Malay	979	75.2
Chinese	98	7.5
Indian	162	12.4
Others (e.g. Kedayan, Bajau, Bangladesh, Indonesia)	63	4.8
**Nationality**		
Malaysian	1275	97.9
Others (e.g. Bangladesh, Indonesia, Mexico)	26	2.1
**Marital status**		
Single/unmarried	1276	98.0
Married	26	2.0
**Level of education**		
Diploma	373	28.6
Degree	849	65.2
Masters	72	5.5
PhD/DrPH	8	0.6
**Household income (MYR/Month), n=1116**		
≤2000	273	24.5
2001–4000	307	27.5
4001–6000	244	21.9
6001–8000	99	8.9
8001–10000	106	9.5
≥10001	87	7.8
**Presence of comorbidity disease**		
None (healthy)	1199	92.1
Asthma	64	4.9
High cholesterol	13	1.0
High blood pressure	7	0.5
Diabetes mellitus	2	0.2
Kidney problem	3	0.2
Heart disease	4	0.3
Others (e.g Allergic, Migraine, Gastric, Cancer)	21	1.6

### Smoking and e-cigarette use profile

**Figure 1:**
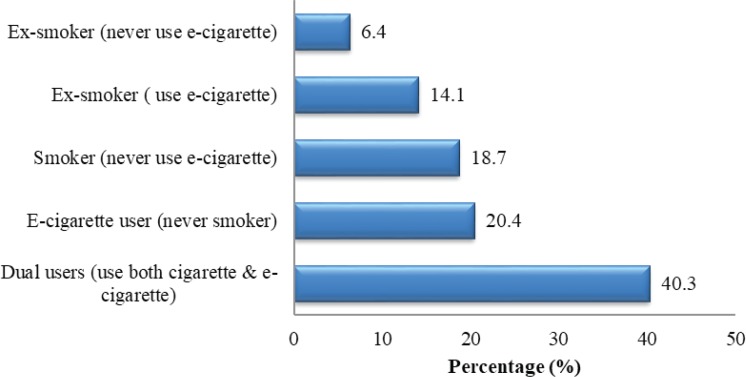
Cigarette/e-cigarette users among students (n=1302 )

Within our sample population of students, 34.5% were exclusive e-cigarette users, and 40.3% were dual users, indicating that 74.8% of the respondents were current e-cigarette users (Figure 1). Moreover, 18.7% were current smokers and 6.4% neither smoked nor used e-cigarettes. Among current e-cigarette users, 40.3% used cigarettes and e-cigarettes simultaneously. In all, 57.5% (749) of the respondents were introduced to e-cigarettes by colleagues in the university, 37.5% (488) by friends outside the university, 36.6% through the internet (websites, social networking sites, blogs, and e-mails) and 28.8% through the communication media (television, radio, mail, print, billboards and outdoor advertisement). A propotion of 37.9% of the respondents bought e-cigarette products from retailers, 17.6% from their fellow university students, and 14.8% online.

Findings in this study revealed that most of the e-cigarette users preferred locally produced e-liquid (55.6%) rather than imported e-liquids (44.4%). A total of 312 (32%) respondents preferred e-liquid that contained ≤6 mg nicotine, followed by 212 (21.7%) who were oblivious to the nicotine level (Table 2). About 178 (18.3%) preferred e-liquid without nicotine, while only 2.4% preferred e-liquid with a high amount of nicotine (≥16 mg).

**Table 2:** Respondents smoking/e-cigarette-use profile

*Profiles*	*Frequency (n)*	*Per cent (%)*
**Age of smoking initiation (years), n=1036**		
<10	39	3.8
10–15	380	36.7
16–20	569	54.9
21–25	48	4.6
**Family member who smokes, n=1284**		
Yes	679	53.5
No	605	46.5
**Average cigarettes per day, n=1036**		
Did not answer	36	3.5
≤5	584	56.4
6–10	252	24.3
11–15	76	7.3
16–20	70	6.8
≥ 21	18	1.8
**Preferable cigarette, n=1036**		
Standard	791	76.4
Light	279	26.9
Menthol	218	21.0
Tobacco (Indonesia)	151	14.6
Cigar/Curut	55	5.3
Others (e.g. Harvest, John, U2, Mevius)	37	3.6
**Source of e-cigarette information**		
Family	100	7.7
Product seller	160	12.3
Advertisement	189	14.5
University student	749	57.5
Internet	476	36.6
Magazines	80	6.1
Outside peer	488	37.5
Media	349	28.8
Unsure	127	9.8
**Source of e-cigarettes**		
Wholesale	109	8.4
Booth seller	135	10.4
Family member	32	2.5
Retailer	493	37.9
Online	193	14.8
Exchange among peers	126	9.7
University student	229	17.6
Outside peers	144	11.1
Unsure	187	14.4
**Number of e-cigarette devices, n=506**		
1	387	76.5
2	81	16.0
3	21	4.2
≥ 4	17	3.6
**Preferable e-juice, n=975**		
Local	542	55.6
Imported	433	44.4
**Types of e-juice, n=975**		
Without nicotine (no flavours)	178	18.3
Without nicotine (with flavours)*	85	8.7
≤6 mg nicotine	312	32.0
≤9 mg nicotine	84	8.6
≤12 mg nicotine	79	8.1
≥16 mg nicotine	23	2.4
Unsure	212	21.7
Unanswered	2	0.2

* e.g. mango, mint, redbull, grape, vanilla.

This study showed that over 67% of the respondents did not report any health impact and were not certain if they suffered any adverse effects from the use of e-cigarettes. Among the adverse effects experienced by the users were: dizziness 14.4%, cough 14.1%, headache 12.4%, addiction to e-cigarette use 9.5%, chest pain 6.9%, and shortness of breath 5.7%. Other adverse effects, experienced by less than 5% of the respondents, were: vomiting 4.5%, decreased appetite 4.1%, insomnia 4.0%, weight loss 1.9%, depression 1.6%, and anxiety 1.1%. Dual users had significantly experienced more adverse effects compared to exclusive e-cigarette users (Table 3).

**Table 3:** Adverse events occurrence based on types of cigarette/e-cigarette users

*Variable*	*Types of cigarette/e-cigarette users (n, %)*						
	*Dual user*	*E-cigarette user (never smoker)*	*E-cigarette user (ex-smoker)*	*Smoker (never use e-cigarette)*	*Ex-smoker (never use e-cigarette)*	*χ2*	*p*
**Adverse events**							
None	302 (33.0)	175 (19.1)	111 (12.1)	244 (26.7)	83 (9.1)	193.488	<0.001*
1 Symptom	103 (54.5)	47 (24.9)	39 (20.6)	0 (0.0)	0 (0.0)		
2 Symptoms	57 (58.8)	21 (21.6)	19 (19.6)	0 (0.0)	0 (0.0)		
≥3 Symptoms	63 (62.4)	23 (22.8)	15 (14.9)	0 (0.0)	0 (0.0)		
Total	525 (40.3)	266 (20.4)	184 (14.1)	244 (18.7)	83 (6.4)		

* Chi-squared test. Values are expressed as frequency and per cent (n, %). p<0.05 compares dual users, e-cigarette user (never smoker), e-cigarette user (ex-smoker), smoker (never use e-cigarette) and ex-smoker (never use e-cigarette).

**Figure 2:**
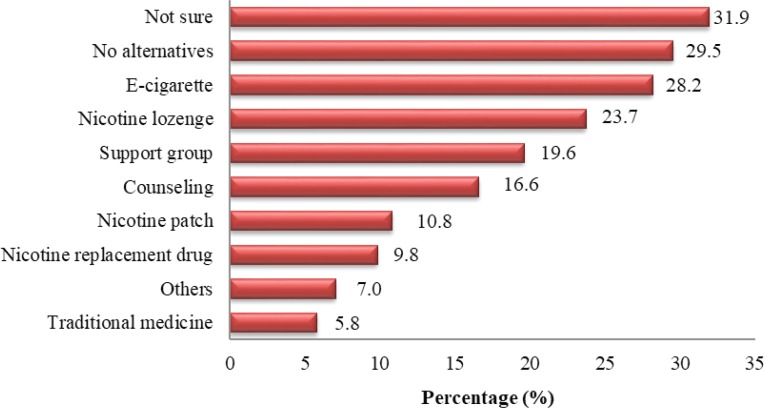
Students opinion on the most effective alternative for smoking cessation (n=1036 )

Figure 2 depicts the feelings of respondents towards e-cigarette use as an alternative to smoking cessation, where 31.9% felt uncertain about the best alternative means for quitting smoking, 29.5% did not mention any need for an alternative, while 28.2% agreed that e-cigarettes could be used as an alternative means for quitting smoking. Further, smoking cessation methods chosen were: nicotine lozenges 23.7%, a support group 19.6% , health counseling 16.6%, nicotine patch 10.8%, nicotine replacement drug 9.8%, other 7.0%, and traditional medicine 5.8%.

With regards to the reason for e-cigarette use provided by dual users (n=975), 64.6% of the respondents use e-cigarette due to their own desire, personal beliefs 64.6%, social influence 54.8%, emotional factors (boredom, loneliness and stress) 62.6%, current trends 62.7%, while 57.8% claimed to use e-cigarettes as a means for quitting smoking.

The study found that majority of the respondents were dual users, the high-risk group (involved in tobacco/nicotine use). The bivariate analysis revealed. that gender as well as three reasons for e-cigarette use (own desire, personal belief, aim to quit smoking) have a significant relationship with e-cigarette use (Table 4). Of the 975 male e-cigarette users, 52.9% were dual users, while 42.3% from the same category of male respondents were exclusive e-cigarette users. Among the dual users’ category, the reasons given for e-cigarette use were: own desire 37.8%, personal belief 37.2%, mood disorder 34.8%, aim to quit smoking 34.4%, current trend 33.8%, and social influence 29.6%. Among the exclusive e-cigarette users’ category, main reasons given for e-cigarette use were: current trend 28.8%, mood disorder 27.8%, personal belief 27.3%, own desire 26.8%, social influence 25.1%, and aim to quit smoking 23.5%.

**Table 4:** Association between dual user and e-cigarette (e-cig) users with sociodemographics, adverse events occurrence, comorbidity disease and reason for e-cigarette use initiation among students (n=975 )

*Variable*	*Dual user n (%)*	*E-cig user n (%)*	*χ2*	*p*	*OR*	*95% CI*	
						*Lower*	*Upper*
**Institution**							
Public	277 (28.4)	226 (23.2)	0.63	0.43	1.11	0.86	1.42
Private	248 (25.4)	224 (23.0)					
**Age (years)**							
≤ 21	320 (32.8)	296 (30.4)	2.42	0.12	0.81	0.62	1.05
> 21	205 (21.0)	154 (15.8)					
**Gender**							
Male	516 (52.9)	412 (42.3)	23.92	<0.001*	0.19	0.09	0.40
Female	9 (0.9)	38 (3.9)					
**Ethnicity**							
Malay	400 (41.0)	350 (35.9)	0.34	0.56	1.09	0.81	1.48
Others	125 (12.8)	100 (10.3)					
**Marital status**							
Single	516 (52.9)	441 (45.2)	0.11	0.74	0.85	0.34	2.17
Married	9 (0.9)	9 (0.9)					
**Level of education**							
Degree	351 (36.0)	280 (28.7)	2.28	0.13	0.82	0.63	1.06
Others	174 (17.8)	170 (17.4)					
**Household income (MYR/month)**							
≤ RM4000	228 (27.8)	206 (25.1)	0.01	0.93	0.99	0.75	1.30
> RM4000	204 (24.9)	182 (22.2)					
**Adverse events occurrence**							
≤ 2 symptoms	462 (47.4)	412 (42.3)	3.30	0.07	0.68	0.44	1.03
>2 symptoms	63 (6.5)	38 (3.9)					
**Comorbidity disease**							
None (healthy)	484 (49.6)	407 (41.7)	0.94	0.33	1.25	0.80	1.95
Having comorbidity	41 (4.2)	43 (4.4)					
**Reason for e-cigarette use**							
**i) Own desire**							
Low	156 (16.0)	189 (19.4)	16.00	<0.001*	0.58	0.45	0.76
High	369 (37.8)	261 (26.8)					
**ii) Social influence**							
Low	236 (24.2)	205 (21.0)	0.04	0.85	0.98	0.76	1.26
High	289 (29.6)	245 (25.1)					
**iii) Personal belief**							
Low	162 (16.6)	184 (18.9)	10.65	<0.001*	0.64	0.50	0.84
High	363 (37.2)	266 (27.3)					
**iv) Currents trends**							
Low	195 (20.0)	169 (17.3)	0.02	0.89	0.98	0.76	1.27
High	330 (33.8)	281 (28.8)					
**v) Aim to quit smoking**							
Low	190 (19.5)	221 (22.7)	16.59	<0.001*	0.59	0.45	0.76
High	335 (34.4)	229 (23.5)					
**vi) Self emotion**							
Low	186 (19.1)	179 (18.4)	1.96	0.16	0.83	0.64	1.08
High	339 (34.8)	271 (27.8)					

Data exclude non-e-cigarette user with analysis using chi-squared test. E-cigarette user refers to exclusive e-cigarette user. OR: odds ratio, CI: confidence interval.

* p<0.05 indicates significant difference does exist.

Logistic regression was performed to ascertain the impact of age, gender, ethnicity, level of education, adverse events occurrence, comorbidity disease and the reason for e-cigarette use. Exclusive e-cigarette use was significantly associated with gender (AOR=0.19, 95% CI: 0.09–0.40, p<0.001) and own desire (AOR=0.58, 95% CI: 0.45–0.76, p<0.001), personal belief (AOR=0.64, 95% CI: 0.50–0.84, p=0.001) and aim to quit smoking (AOR=0.59, 95% CI: 0.45–0.76, p<0.001). However, the multivariate analysis showed that exclusive e-cigarette use was only associated with gender (p<0.001) with an adjusted odds ratio of becoming e-cigarette user 0.18 times lower for males compared to females (Table 5).

**Table 5:** Factors associated with e-cigarette users in univariate and multivariate analysis among students (n=975 )

*Variable*	*Univariate analysis*			*Multivariate analysis*		
	*0=dual user, 1=e-cigarette user (reference)*					
	*p*	*AOR*	*95% CI*k	*p*	*AOR*	*95% CI*
**Age (years)**						
≤ 21 (reference)	0.12	0.81	0.62–1.05	0.17	0.82	0.62–1.09
>21						
**Gender**						
Female (reference)	<0.001*	0.19	0.09–0.40	<0.001*	0.18	0.09–0.39
Male						
**Ethnicity**						
Others (reference)	0.56	1.09	0.81–1.48	0.17	1.24	0.91–1.71
Malay						
**Level of education**						
Others (reference)	0.13	0.82	0.63–1.06	0.44	0.89	0.67–1.19
Degree						
**Adverse events occurrence**						
≤ 2 symptoms (reference)	0.07	0.68	0.44–1.03	0.05	0.65	0.42–1.01
>2 symptoms						
**Comorbidity disease**						
None (reference)	0.33	1.25	0.80–1.9	0.29	1.29	0.81–2.05
Having comorbidity						
**Reason for e-cigarette use**						
**i) Own desire**						
Low (reference)	<0.001*	0.58	0.45–0.76	0.27	0.82	0.59–1.16
High						
**ii) Personal belief**						
Low (reference)	0.001*	0.64	0.50–0.84	0.34	0.85	0.61–1.19
High						
**iii) Aim to quit smoking**						
Low (reference)	<0.001*	0.59	0.45–0.76	0.06	0.72	0.51–1.01
High						

Data exclude non-e-cigarette user with analysis using logistic regression. E-cigarette user refers to exclusive e-cigarette user. AOR: adjusted odds ratio, CI: confidence interval. *p<0.05 indicates significant difference does exist.

## DISCUSSION

The study assessed factors related to e-cigarette use through which user profiles were created. The study revealed that male respondents were the majority e-cigarette users, most of them young and residing in urban areas. The respondents smoked cigarettes alongside their family members, colleagues and peers who either smoked tobacco, used an e-cigarette, or both. This finding is similar to the e-cigarette user profile reported by Jun et al.^20^ and Joan-Carles et al.^21^. In Malaysia, smoking among females is culturally unacceptable, which affirms the low number of female e-cigarette users found in the study. Young people have strong desires to try something new; living in an urban settings allows easy access to e-cigarette sources and living in proximity to a smoking environment enhances smoking interest. Involvement with e-cigarette use exposes the users to various health risks, particularly those who use it as an alternative to quitting cigarette smoking. The implication here is the possible lack of knowledge about smoking quitting methods, despite the availability of alternative means for quitting cigarette smoking, such as nicotine lozenges, support group, counselling, nicotine patch, nicotine replacement drug and traditional medicine. The listed methods are safe and subsidized by the government, but were only used by 25% of the respondents.

In this study, about 74.8% of the respondents were e-cigarette users, the finding is augmented by the Saddleson et al.^36^ assertion that most university/college students use e-cigarettes for pleasure. However, a study conducted in France found that 70% of college students in two major campuses never used e-cigarette^37^. Also, some studies in the United States and other countries show that e-cigarette users may as well use conventional cigarettes^38,39^ and drugs^40^ in the future. It is also worrying that e-cigarette use among ex-smokers may cause a return to cigarette smoking^41^.

Over three-quarters of the respondents were involved in smoking, including ex-smokers. Of them, 54.9% started smoking at the age of 16–20 years, when they were in high school or just entering university. This situation clearly indicates that strategic prevention planning should target this age group and younger. Rigotti^42^ stated that university students should be targeted for behavior change advocacy, because adolescents were the main marketing target group of the tobacco industry. It was further revealed that 34.5% of the respondents were exclusive e-cigarette users, with preference to e-juice without nicotine (only flavors) and lower level of nicotine (≤ 6 mg of nicotine)^41^.

Health effects of smoking are well known due to the existence of evidence-based research outcomes and established linkage to cancer, heart disease, and stroke. Information on smoking hazards are available and easily accessible through various media outlets (e.g. posters, advertisements on buildings and vehicles, video, radio and television ads etc.). Despite this development for a conventional cigarette, information on e-cigarette adverse effects are relatively new and emerging, and in need of more research. In this study, adverse effects like dizziness, cough, headache, addiction and chest pain were reported. Findings by Hua et al.^43^ posit that there are over 405 different health-related effects experienced by users. Additionally, most common health-related effects occurred in the respiratory, neurological, sensory and digestive systems, while direct health effects occurred solely in the respiratory system^43^.

E-cigarette marketers often advertise it as a safe and healthier alternative to conventional smoking and that it aids smoking cessation^44-48^. The study found that e-cigarettes were used by the respondents for various reasons: own desire, personal beliefs, self-emotion, and as a current smoking trend. Using it as a means for quitting smoking is popular among dual users (59.4%). Chapman et al.^49^ stated that e-cigarette use is not consistent with attempting to quit tobacco smoking among young adults, as adults most often report e-cigarette use as a substitute for tobacco and not as a means for quitting^24,25,49^.

The effectiveness of e-cigarette use as a smoking cessation tool is unclear^1,4,20^, and subject for further research. Moreover, e-cigarette use is not without risk, but much less dangerous than tobacco, as it has less carcinogenic chemicals (e.g. acetone, acroline, benzene, cadmium, carbon monoxide, toluene, etc.)^46,48^. Lynn et al.^50^ suggested that the mistaken perception of lesser risk may be the influencing factor for e-cigarette use as a substitute for tobacco smoking. Further research on the health effects of e-cigarette use should be conducted, to ascertain its severity on human health.

The main limitation of this study is that the data were only collected in one of the States in Malaysia within a certain time duration. Therefore, the samples do not represent a national proportion and hence have limited generalizability. Also, the self-reporting mechanism used in data collection could result in a biased response. Despite these limitations, the findings present a good profile of e-cigarette users and a framework with which to guide decision making and future research on e-cigarette use. Additionally, e-cigarette use was more popular among young people in this study, larger studies in diverse locations and populations might address the challenges of this study.

## CONCLUSIONS

The study provides evidence-based information for a behaviour change campaign on e-cigarette use among university students. Both cigarettes, as well as e-cigarette use among students, for whatever reason, need to be discouraged. Achieving meaningful mitigation of the use of tobacco products needs the combined efforts of academics, civil society, government, industry and communities to find effective means for a solution to the tobacco epidemic.
